# Bridging the Knowledge–Practice Gap in Breast Cancer Awareness: A Nationwide Online Cross‐Sectional KAP Study Among Adult Females in Nigeria

**DOI:** 10.1002/hsr2.73231

**Published:** 2026-09-13

**Authors:** Madinat Hassan, Taofeek Tope Adegboyega, Bilkisu Usman Farouk, Sunday Zeal Bala, Rachael Olufunmilayo Oduyemi, Kazeem Owolabi, Munir Sani, Miracle Uwa Livinus, Hannatu Usman Ayuba, Sadiq Hassan, Ifeyinwa Maureen Okeke, Abednego Shekari, Murtala Jibril

**Affiliations:** ^1^ Foresight Institute of Research and Translation (FIRAT) Kigali Rwanda; ^2^ Department of Pharmacology, School of Medicine and Health Sciences, CMHS University of Rwanda Kigali Rwanda; ^3^ Biology Unit, Faculty of Science Air Force Institute of Technology (AFIT) Kaduna Kaduna State Nigeria; ^4^ Khayr Cancer Health Initiative (KCHI) Kaduna Kaduna State Nigeria; ^5^ Department of Microbiology and Parasitology, CMHS University of Rwanda Kigali Rwanda; ^6^ Department of Radiology Barau Dikko Teaching Hospital (BDTH) Kaduna Kaduna State Nigeria; ^7^ Department of Pharmaceutical Sciences University of Maryland Eastern Shore Princess Anne Maryland USA; ^8^ NorthCare Hospital (NCH) Kaduna Kaduna State Nigeria; ^9^ Faculty of Nursing Chrisland University Abeokuta Ogun State Nigeria; ^10^ Department of Radiology Ahmadu Bello University (ABU) Zaria Kaduna State Nigeria; ^11^ Department of Biochemistry, School of Sciences and Information Technology Skyline University Kano Kano State Nigeria; ^12^ Department of Radiation and Clinical Oncology National Hospital Abuja Nigeria; ^13^ Department of Community Medicine Alex Ekwueme Federal University Abakaliki Ebonyi State Nigeria; ^14^ Department of Chemistry and Biochemistry Old Dominion University Norfolk Virginia USA; ^15^ African Centre for Disease Control (ACDC) Abuja Nigeria; ^16^ Enabel Addis Ababa Ethiopia

**Keywords:** awareness, breast cancer, breast self‐examination, early detection, Nigeria, screening

## Abstract

**Background and Aims:**

Breast cancer (BCa) is a major cause of cancer morbidity and mortality among women in Nigeria, with late presentation contributing to poor outcomes. This study assessed knowledge, attitudes, and willingness to perform breast self‐examination (BSE) among adult females in Nigeria and examined associated factors.

**Methods:**

We conducted a descriptive cross‐sectional online survey between February 16 and April 30, 2024. A structured, pretested questionnaire was disseminated through digital platforms, yielding 711 consenting adult female respondents from all six geopolitical zones. Knowledge, attitude, and willingness‐to‐perform‐BSE measures were analyzed using descriptive statistics, linear regression, and binary logistic regression. Associations were assessed using two‐sided tests with α = 0.05. Because recruitment was online and non‐probability based, the sample should not be interpreted as nationally representative.

**Results:**

Of 711 respondents, 692 (97.3%) knew what BCa was, whereas 259 (36.4%) were aware of early screening modalities, and 321 (45.1%) reported knowledge of the BSE procedure. Positive attitudes toward screening were common: 704 (98.9%) considered screening useful for early detection, and 691 (97.3%) believed early screening improves survival. Although 696 (97.9%) reported willingness to perform BSE after instruction, this represented intention rather than verified regular or technically correct practice. Higher educational attainment was associated with greater willingness to perform BSE, but estimates were imprecise because the basic‐education reference group was very small. Reliance on social media, radio/television, and campaigns was associated with lower knowledge scores, although these observational associations do not establish causality, and participants could report multiple information sources.

**Conclusion:**

Among this digitally connected and relatively highly educated sample of adult Nigerian females, general awareness and attitudes toward BCa screening were favorable, but detailed knowledge of risk factors, screening modalities, and BSE procedures remained limited. Public health interventions should move beyond awareness creation to provide accurate, skill‐based education through trusted channels. The findings should not be generalized to all Nigerian women, particularly those who are rural, less educated, older, or have limited digital access.

## Summary

- High general awareness of breast cancer coexisted with substantial gaps in actionable knowledge, particularly regarding risk factors, symptoms, treatment, and screening options.
- A marked discrepancy was identified between willingness to perform breast self‐examination (BSE) and knowledge of the correct BSE procedure, highlighting an important knowledge–intention gap.
- Higher educational attainment was associated with greater willingness to perform BSE but not necessarily with higher breast cancer knowledge, suggesting that formal education alone may not ensure health literacy.
- Digital and mass‐media platforms were major sources of breast cancer information, but information‐source exposure alone was not consistently associated with better knowledge.
- The findings support a shift from broad awareness campaigns toward practical, skill‐based, and context‐specific breast cancer education that promotes actionable early‐detection behaviors.

## 1 Introduction

Breast cancer (BCa) is a major public health problem and remains one of the most commonly diagnosed cancers among women worldwide. Despite advances in screening and treatment, late presentation continues to contribute substantially to BCa morbidity and mortality, particularly in low‐ and middle‐income countries, including Nigeria [1, 2, 3]. Delayed diagnosis is influenced by multiple factors, including limited awareness of BCa symptoms and risk factors, misconceptions, fear, sociocultural beliefs, and barriers to accessing appropriate healthcare [4, 5, 6, 7, 8].

Awareness of BCa is an important component of early detection. However, general awareness does not necessarily translate into comprehensive knowledge or appropriate preventive behavior. Women may recognize BCa as a health problem while having limited knowledge of its risk factors, warning signs, available screening modalities, or the correct technique for breast self‐examination (BSE) [9, 10, 11, 12, 13]. Similarly, willingness or intention to perform BSE should not be equated with regular or technically correct practice. The transition from knowledge and intention to sustained preventive behavior may be influenced by self‐efficacy, perceived risk and benefits, social norms, access to information, and practical barriers [14, 15, 16].

Previous studies in Nigeria have reported considerable variation in BCa knowledge, attitudes, and BSE‐related behaviors among women [5, 7, 13, 17]. Although some studies have demonstrated high awareness and favorable attitudes towards BCa screening, others have identified substantial gaps in knowledge of symptoms, risk factors, and screening methods [7, 13, 18]. Differences between studies may reflect variations in study populations, geographical settings, educational attainment, access to health information, and the measurement of BSE outcomes. In particular, many studies have focused on whether women have ever practiced BSE, while less attention has been given to the distinction between general awareness, procedural knowledge, and willingness to engage in BSE.

The increasing use of digital platforms has further transformed the ways in which women obtain health information. Social media, the internet, television, radio, health campaigns, and healthcare facilities may provide important opportunities to improve BCa awareness. However, exposure to information does not necessarily guarantee access to accurate, complete, or actionable knowledge. The quality and content of information may vary considerably across sources, and women may obtain information from multiple channels. Consequently, understanding the relationship between information sources, knowledge, perceptions, and willingness to engage in preventive behaviors is important for designing effective BCa education strategies.

Despite the growing literature on BCa awareness in Nigeria, important gaps remain in understanding whether broad awareness translates into more specific knowledge and willingness to engage in early‐detection behaviors. This study therefore assessed BCa knowledge, perceptions, information sources, and willingness to perform BSE among adult females in Nigeria. By examining these domains together, the study sought to identify potential gaps between awareness, actionable knowledge, and preventive intention and to generate evidence to inform targeted BCa awareness and early‐detection interventions.

## 2 Methodology

### 2.1 Study Design

A descriptive cross‐sectional survey was conducted across the six geopolitical zones of Nigeria to explore the knowledge, attitudes, and practices (KAP) of Nigerian adult women regarding BCa and BSE. This design was selected because it enables the collection and analysis of relevant data at a single point in time, offering a snapshot of the prevailing awareness and behavioral tendencies within the population.

### 2.2 Study Area and Population

The study targeted Nigerian adult females aged 18 years and above residing in the six geopolitical zones of Nigeria. Eligible participants were Nigerian citizens who could read and comprehend English and were willing to provide informed consent. Because recruitment was conducted through an online English‐language questionnaire disseminated via digital platforms, participation required internet access and sufficient digital literacy. The sampling strategy was non‐probability and online; therefore, the study population was not a probability sample of all adult Nigerian females. Rural women, older women, women with lower educational attainment, and those with limited internet access were likely to be underrepresented.

### 2.3 Sample Size and Method

The minimum sample size was estimated using the formula described by Fisher et al. [12],

$$n=\frac{Z^{2}\mathrm{pq}}{d^{2}}$$

with *Z* = 1.96 for a 95% confidence level, *p* = 0.20 as the assumed prevalence, *q* = 1–0.20 = 0.8, and *d* = 0.05 as the margin of error from a previous study [12].

The calculated minimum sample size was 246. Recruitment continued beyond this minimum, and 711 adult females provided informed consent and completed the questionnaire. Because the final sample was obtained through online, non‐probability recruitment, the achieved sample size does not confer national representativeness.

### 2.4 Data Collection

Data collection used a structured, self‐administered online questionnaire hosted on Google Forms. The survey link was disseminated through X (formerly Twitter), LinkedIn, Facebook, email, Telegram, WhatsApp, and other digital channels. This approach enabled a broad geographic reach but introduced the possibility of selection bias related to internet access, digital literacy, education, age, and urban residence.

### 2.5 Data Collection Tool

Data collection occurred from February 16 to April 30, 2024, using a structured and pretested questionnaire administered through Google Forms.

### 2.6 Validity and Reliability

The instrument was adapted from previously published BCa KAP tools [13, 14] and refined through expert review and pretesting. A pilot study was conducted among 24 adult women with BCa (10% of the sample size) outside the study site to test clarity and reliability. Reliability was assessed using Cronbach's alpha, with a threshold of ≥ 0.78. The questionnaire comprised five sections: demographics; knowledge of BCa risk factors, symptoms, screening, and treatment; information sources; willingness to perform BSE and related procedural knowledge; and attitudes toward BCa screening. The online format and English‐language administration were considered when interpreting the representativeness of the sample.

Knowledge of BCa was assessed using factual items covering awareness of the disease, causes, risk factors, signs and symptoms, screening methods, treatment options, and BSE procedures. Dichotomous items were scored 1 for a correct response and 0 for an incorrect or “not sure” response. For multiple‐response items, respondents received credit when they selected at least one correct option. A composite knowledge score was generated by summing correct responses. For descriptive and regression analyses, a score of ≥ 5 correct responses was classified as adequate knowledge; this threshold represented at least 50% of the assessed knowledge items and was used as an analytical categorization rather than a validated diagnostic cut‐off.

Attitudes toward BCa screening were assessed using five Likert‐type statements addressing perceived usefulness of screening, survival benefits of early detection, the importance of BSE, preference for female health workers, and misconceptions regarding the effect of early screening on treatment. Cronbach's alpha for the attitude scale was 0.78, indicating acceptable internal consistency. Negatively worded items were reverse‐coded so that higher scores indicated more favorable attitudes. The attitude scale showed acceptable internal consistency in the original analysis; a Cronbach's alpha value was not available in the dataset used for this revision and is therefore not reported.

Practice‐related outcomes were analyzed as distinct constructs. First, willingness to perform BSE was assessed by asking whether participants would practice BSE after being taught. Second, procedural knowledge relevant to technically correct BSE was assessed using items addressing awareness of the procedure, age of initiation, timing in relation to menstruation, and who should perform the examination. The study did not directly measure observed, regular, or technically verified BSE performance. Accordingly, the term “willingness to perform BSE” is used throughout when referring to the intention outcome, while procedural knowledge is reported separately.

### 2.7 Data Analysis

Data were analyzed using SPSS version 21.0. Categorical variables were summarized using frequencies and percentages, and continuous knowledge and attitude scores were analyzed as continuous outcomes. Linear regression was used to examine associations between prespecified demographic predictors and continuous knowledge and attitude scores. Binary logistic regression was used for dichotomous outcomes, including willingness to perform BSE and adequate knowledge.

Associations between knowledge category and willingness to perform BSE were also examined. Multiple‐response information‐source items were analyzed according to the number of respondents selecting each source; therefore, percentages may not sum to 100%. All inferential tests were two‐sided, and statistical significance was set a priori at α = 0.05. *p* values were interpreted alongside effect estimates and their precision, where available. Because some predictor categories were sparse, regression analyses were considered exploratory and hypothesis‐generating rather than causal or definitive. Large coefficients, odds ratios, and standard errors were interpreted cautiously as possible evidence of data sparsity or quasi‐complete separation.

## 3 Results

### 3.1 Demographic and Background Characteristics of Respondents

Table 1 presents the characteristics of the 711 adult female respondents. Participants were recruited from all six geopolitical zones, although representation was uneven. The Northwest contributed 172 (24.2%) respondents and the Southeast 86 (12.1%). Most respondents were aged 26–35 years (279/711, 39.2%), lived in urban areas (594/711, 83.5%), and had postgraduate education (459/711, 64.6%). The sample was therefore predominantly urban and highly educated. More than half were married (378/711, 53.2%) and employed (404/711, 56.8%).

**Table 1:** Demographic and background characteristics of adult female respondents (*N* = 711).

Characteristic	Category	Frequency	Percentage (%)
Geographical zone	Northwest	172	24.2
	North central	142	20
	Southwest	113	15.9
	South‐south	100	14.1
	North east	98	13.8
	Southeast	86	12.1
Age groups	26‐35 years	279	39.2
	18‐25 years	180	25.3
	36‐45 years	147	20.7
	46+ years	105	14.8
Religion	Islam	469	66
	Christianity	241	33.9
	Agnostic	1	0.1
Settlement type	Urban	594	83.5
	Rural	117	16.5
Education level	Postgraduate	459	64.6
	Undergraduate	185	26
	Secondary education	57	8
	Basic education	6	0.8
	Not applicable	4	0.6
Marital status	Married	378	53.2
	Single	283	39.8
	Widowed	32	4.5
	Divorced	18	2.5
Occupation	Employed	404	56.8
	Unemployed	166	23.3
	Self‐employed	133	18.7
	Retired	8	1.1
Food habit	Non‐vegetarian	578	81.3
	Vegetarian	133	18.7

### 3.2 BCa Knowledge Assessment

Among the 711 respondents, the most frequently recognized risk factor was a family history of BCa (384/711, 54.0%), followed by alcohol and tobacco consumption (243/711, 34.2%), obesity (213/711, 30.0%), physical inactivity (202/711, 28.4%), fatty‐food consumption and stressful life (188/711 each, 26.4%). Recognition of radiation exposure (183/711, 25.7%), hormone replacement therapy (140/711, 19.7%), contraceptive use (123/711, 17.3%), and reproductive history (42/711, 5.9%) was less common (Table 2).

**Table 2:** Recognition of BCa risk factors among respondents (*N* = 711).

Risk factor	Frequency	Percentage (%)
Family history of BCa	384	54
Alcohol and tobacco consumption	243	34.2
Obesity	213	30
Physical inactivity practice	202	28.4
Consumption of fatty foods	188	26.4
Stressful life	188	26.4
Exposure to radiation	183	25.7
Hormone replacement therapy	140	19.7
Use of contraceptives	123	17.3
Reproductive history	42	5.9
Depression	24	3.4
Breastfeeding	18	2.5

### 3.3 Knowledge Indicators

**Figure 1:**
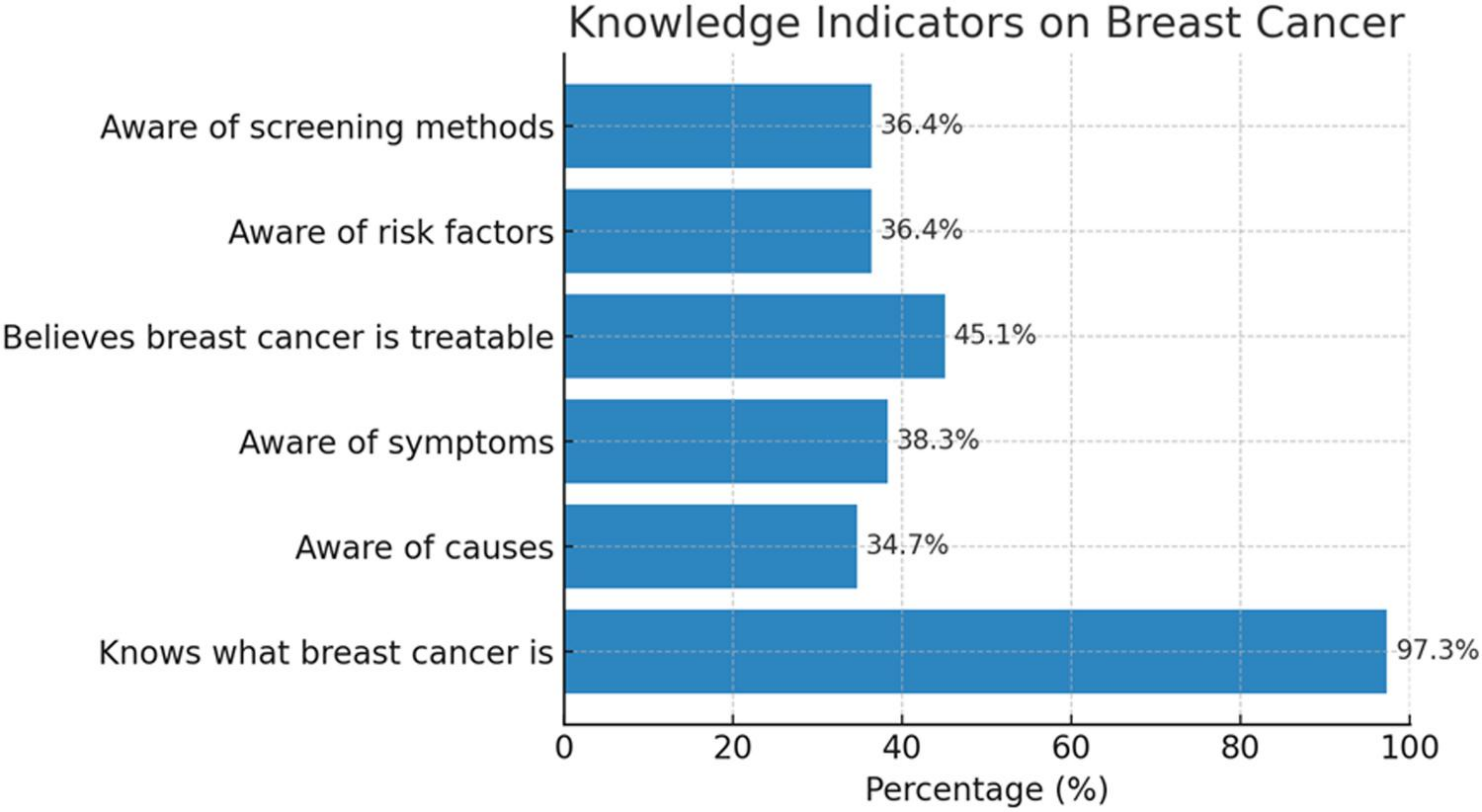
Distribution of knowledge indicators among study participants.

Among the 711 respondents, 692 (97.3%) knew what BCa was, but fewer identified its causes (247/711, 34.7%), signs and symptoms (272/711, 38.3%), or potential risk factors (259/711, 36.4%). Only 321 (45.1%) believed that BCa was treatable. Thus, broad recognition of BCa was high, whereas more detailed knowledge required for informed prevention and early detection was less complete (Figure 1).

Table 3 shows that general recognition of BCa was high, but knowledge of causes, signs and symptoms, treatment, and risk factors was substantially less complete.

**Table 3:** General BCa knowledge indicators among respondents (*N* = 711).

Knowledge indicator	Yes	No
	*N* (%)	*N* (%)
Know what BCa is	692 (97.3)	19 (2.7)
Aware of the causes of BCa	247 (34.7)	464 (65.3)
Aware of BCa signs and symptoms	272 (38.3)	439 (61.7)
Believe that BCa is treatable	321 (45.1)	390 (54.9)
Aware of potential risk factors	259 (36.4)	452 (63.6)

Among the 711 respondents, 259 (36.4%) were aware of early screening modalities (Table 4). BSE was identified by 410 (57.7%), clinical breast examination by 183 (25.7%), and mammography by 166 (23.3%). MRI and genetic testing were rarely identified (2/711, 0.3%, and 1/711, 0.1%, respectively).

**Table 4:** BCa screening knowledge among respondents (*N* = 711).

Screening knowledge component	Frequency	Percentage (%)
**Aware of early screening modalities**		
Yes	259	36.4
No	452	63.6
**Screening methods**		
Breast self‐examination (BSE)	410	57.7
Clinical breast examination (CBE)	183	25.7
Mammography	166	23.3
Breast MRI	2	0.3
Genetic Testing	1	0.1

Self‐rated knowledge of treatment varied: 243/711 (34.2%) rated their knowledge as poor, and 84/711 (11.8%) as very poor, whereas 234/711 (32.9%) rated it as good. Surgery (353/711, 49.6%) and chemotherapy (349/711, 49.1%) were the most frequently identified treatment options, followed by radiotherapy (217/711, 30.5%), hormone therapy (35/711, 4.9%), and targeted therapy (30/711, 4.2%) (Table 5).

**Table 5:** BCa treatment knowledge among respondents (*N* = 711).

Treatment knowledge component	Frequency	Percentage (%)
**Self‐reported knowledge level**		
Poor	243	34.2
Good	234	32.9
Very poor	84	11.8
Excellent	47	6.6
Very good	46	6.5
**Treatment options identified**		
Surgery	353	49.6
Chemotherapy	349	49.1
Radiation therapy	217	30.5
Hormone therapy	35	4.9
Targeted therapy	30	4.2

Knowledge of BSE procedures was incomplete. Of 711 respondents, 321 (45.1%) reported awareness of the BSE procedure, while 390 (54.9%) did not. Although 289 (40.6%) identified initiation at puberty and continuation through life, responses regarding age and timing were inconsistent. Only 285 (40.1%) identified the appropriate timing as approximately 1 week after menstruation, while 223 (31.4%) were unsure. A further 525 (73.8%) believed that morning was the best time for BSE, indicating a common misconception (Table 6).

**Table 6:** Knowledge of breast self‐examination procedures among respondents (*N* = 711).

BSE knowledge component	Frequency	Percentage (%)
**Aware of the BSE procedure**		
Yes	321	45.1
No	390	54.9
**Appropriate age to begin BSE**		
Begin at puberty and continue throughout life	289	40.6
20‐40 years	188	26.4
From puberty	151	21.2
Not sure	46	6.5
After menopause	37	5.2
**Who should perform BSE?**		
Individual (self)	334	47
Any combination of the above	198	27.8
Doctor	94	13.2
Trained nurse	71	10
Not sure	14	2
**Best time for BSE**		
A week after the menstrual flow	285	40.1
I am not sure	223	31.4
During menstrual flow	119	16.7
Not sure	49	6.9
During pregnancy	35	4.9
**Timing of BSE**		
Morning	525	73.8
Not sure	186	26.2

### 3.4 Attitude Assessment of BCa

Attitudes toward BCa screening were generally favorable. Of 711 respondents, 704 (98.9%) agreed that screening modalities are useful for early detection, 698 (98.2%) agreed that BSE is important for early diagnosis, and 691 (97.3%) agreed that early screening increases survival chances. A preference for a female health worker for breast examination was reported by 648 (91.1%). However, 142 (20.0%) agreed with the incorrectly worded statement that early screening does not affect treatment (Table 7).

**Table 7:** Attitudes toward BCa screening among respondents (*N* = 711).

Statement	Strongly agree/agree	Not sure	Disagree/strongly disagree
Early screening methods do not affect treatment (reversed)	142 (20.0%)	49 (6.9%)	520 (73.1%)
I prefer a female health worker for breast examination	648 (91.1%)	33 (4.6%)	30 (4.2%)
Early screening increases survival chances	691 (97.3%)	10 (1.4%)	10 (1.4%)
Breast self‐examination is important for early diagnosis	698 (98.2%)	4 (0.6%)	9 (1.3%)
Screening modalities are useful for early detection	704 (98.9%)	—	7 (1.0%)

#### 3.4.1 BCa Screening‐Related Willingness and Responses

**Figure 2:**
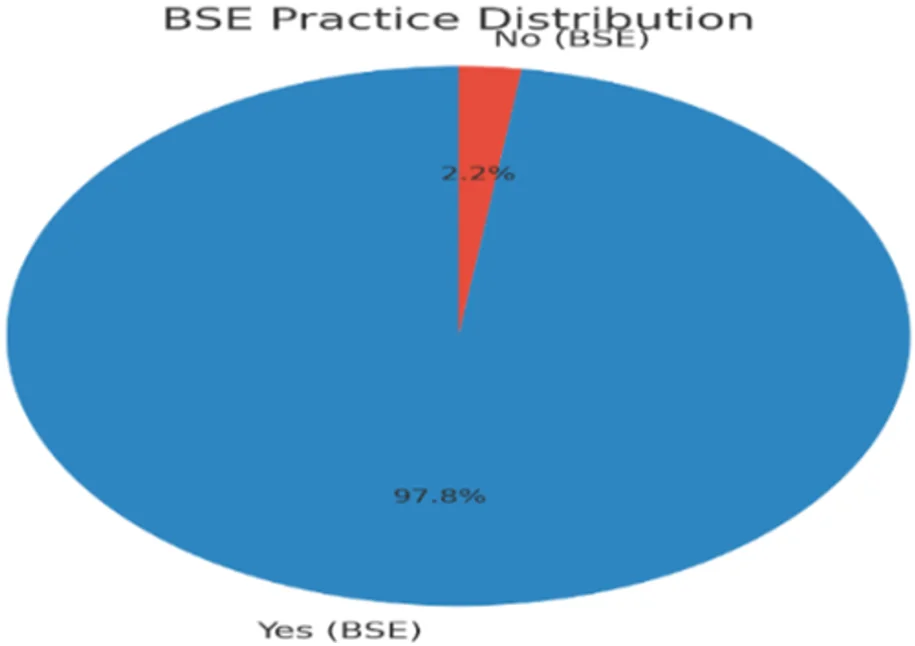
Distribution of breast self‐examination practice willingness.

Nearly all respondents reported willingness to perform BSE after being taught: 696/711 (97.9%) answered yes, while 15/711 (2.1%) answered no. This outcome reflects stated intention rather than verified regular or technically correct BSE performance (Figure 2).

### 3.5 Appropriate Action to Undertake When a Breast Lump Is Discovered

Table 8 shows respondents that noticed a breast lump. Among the 711 respondents, 646 (90.9%) reported that they would visit a doctor at the nearest health center. Smaller proportions would first report the finding to a relative or friend (31/711, 4.4%), visit a traditional doctor (12/711, 1.7%), or were unsure what to do (8/711, 1.1%).

**Table 8:** Intended action if a breast lump is noticed among respondents (*N* = 711).

Action	Frequency	Percentage (%)
Visit a doctor at the nearest health center	646	90.9
Report to a relative/friend	31	4.4
Visit a traditional doctor	12	1.7
Not sure of what to do	8	1.1
Personal prayer	5	0.7
Consult my pastor or an Imam	5	0.7
Keep to yourself and remain unbothered	3	0.4
Keep to yourself to avoid embarrassment	1	0.1
Total	711	100

#### 3.5.1 Socio‐Demographic Associations With Knowledge, Attitudes, and Willingness to Perform BSE

The linear regression model for knowledge scores (Table 9) explained a modest proportion of the observed variance (adjusted R^2^ = 6.5%; F[8,706] = 7.20; *p* < 0.001). Participants aged 46 years or older had slightly lower knowledge scores than those aged 18–25 years (β = −0.125, SE = 0.063; *p* = 0.047). Education coefficients were imprecise and should be interpreted cautiously because the basic‐education reference group was very small (6/711, 0.8%).

**Table 9:** Linear regression model for BCa knowledge score.

Predictor	Estimate	Standard error	*t*‐value	*p*‐value
Age 26–35 (vs. 18–25)	−0.074	0.048	−1.541	0.124
Age 36–45 (vs. 18–25)	−0.039	0.059	−0.668	0.505
Age 46+ (vs. 18–25)	−0.125	0.063	−1.99	0.047*
Secondary education (vs. Basic)	−0.206	0.19	−1.083	0.279
Undergraduate (vs. Basic)	−0.054	0.183	−0.292	0.77
Postgraduate (vs. Basic)	−0.122	0.182	−0.666	0.506
South Region (vs. North)	−0.002	0.034	−0.07	0.944

*Note:* Knowledge score was analyzed as a continuous variable using linear regression. Regression coefficients (β) represent the change in knowledge score per unit change in the predictor variable.

The model for attitude scores (Table 10) explained little of the observed variance (adjusted R^2^ = 1.1%). Although the overall model was statistically significant at the prespecified threshold (F[8,706] = 1.96; *p* = 0.048), its explanatory power was minimal. The only statistically significant coefficient was a modest negative association for respondents aged 46 years or older compared with those aged 18–25 years (β = −0.125, SE = 0.063; *p* = 0.047).

**Table 10:** Linear regression model for attitude toward BCa screening.

Predictor	Estimate	Standard error	*t*‐value	*p*‐value
Age 26–35 (vs. 18–25)	−0.074	0.048	−1.541	0.124
Age 36–45 (vs. 18–25)	−0.039	0.059	−0.668	0.505
Age 46+ (vs. 18–25)	−0.125	0.063	−1.99	0.047*
Secondary education (vs. Basic)	−0.206	0.19	−1.083	0.279
Undergraduate (vs. Basic)	−0.054	0.183	−0.292	0.77
Postgraduate (vs. Basic)	−0.122	0.182	−0.666	0.506
South Region (vs. North)	−0.002	0.034	−0.07	0.944

*Note:* Linear regression was applied. Negatively worded items were reverse‐coded before analysis. Regression coefficients (β) reflect changes in attitude score.

#### 3.5.2 Willingness to Perform BSE and Associated Factors

Binary logistic regression was performed to identify factors associated with respondents’ willingness to perform BSE. As shown in Table 11, higher educational attainment was significantly associated with greater willingness to perform BSE compared with the basic education reference group. In contrast, age, geographical region, overall knowledge score, and attitude score were not significantly associated with willingness to perform BSE.

**Table 11:** Binary logistic regression model for willingness to perform BSE.

Predictors	Estimate	Standard error	Odds ratio	*p*‐value
Secondary education (vs. Basic)	2.776	1.289	16.06	0.031*
Undergraduate (vs. Basic)	4.035	1.207	56.3	0.001***
Postgraduate (vs. Basic)	3.26	1.07	26.05	0.002**
Age 26–35 (vs. 18–25)	−0.164	0.769	0.85	0.831
Age 36–45 (vs. 18–25)	−0.402	0.848	0.67	0.636
South Region (vs North)	0.535	0.58	1.71	0.356
Knowledge score	0.137	0.16	1.15	0.39
Attitude score	0.406	0.551	1.5	0.461

*Note:* Odds ratios were estimated using binary logistic regression. Willingness represents intention rather than demonstrated procedural competence.

#### 3.5.3 Knowledge Level and Willingness to Perform BSE

The relationship between knowledge level and willingness to perform BSE was examined (Table 12). Among respondents with high knowledge scores (≥ 5), 216/219 (98.6%) reported willingness to perform BSE; among those with lower knowledge scores (< 5), 483/496 (97.4%) reported willingness.

**Table 12:** Knowledge level and willingness to perform BSE.

Knowledge level	Willingness to perform BSE	Count	Percentage (%)
High (≥ 5)	Yes	216	98.60%
High (≥ 5)	No	3	1.40%
Low (< 5)	Yes	483	97.40%
Low (< 5)	No	13	2.60%

*Note:* This outcome reflects procedural knowledge required for effective breast self‐examination and was analyzed separately from willingness to practice BSE.

#### 3.5.4 Knowledge as a Predictor of Willingness to Perform BSE

The analysis found little evidence that the knowledge score was associated with willingness to perform BSE. Because willingness was high in both knowledge groups, the outcome distribution was highly imbalanced, and the model had limited ability to discriminate between respondents.

### 3.6 Information Sources and Their Influence

**Figure 3:**
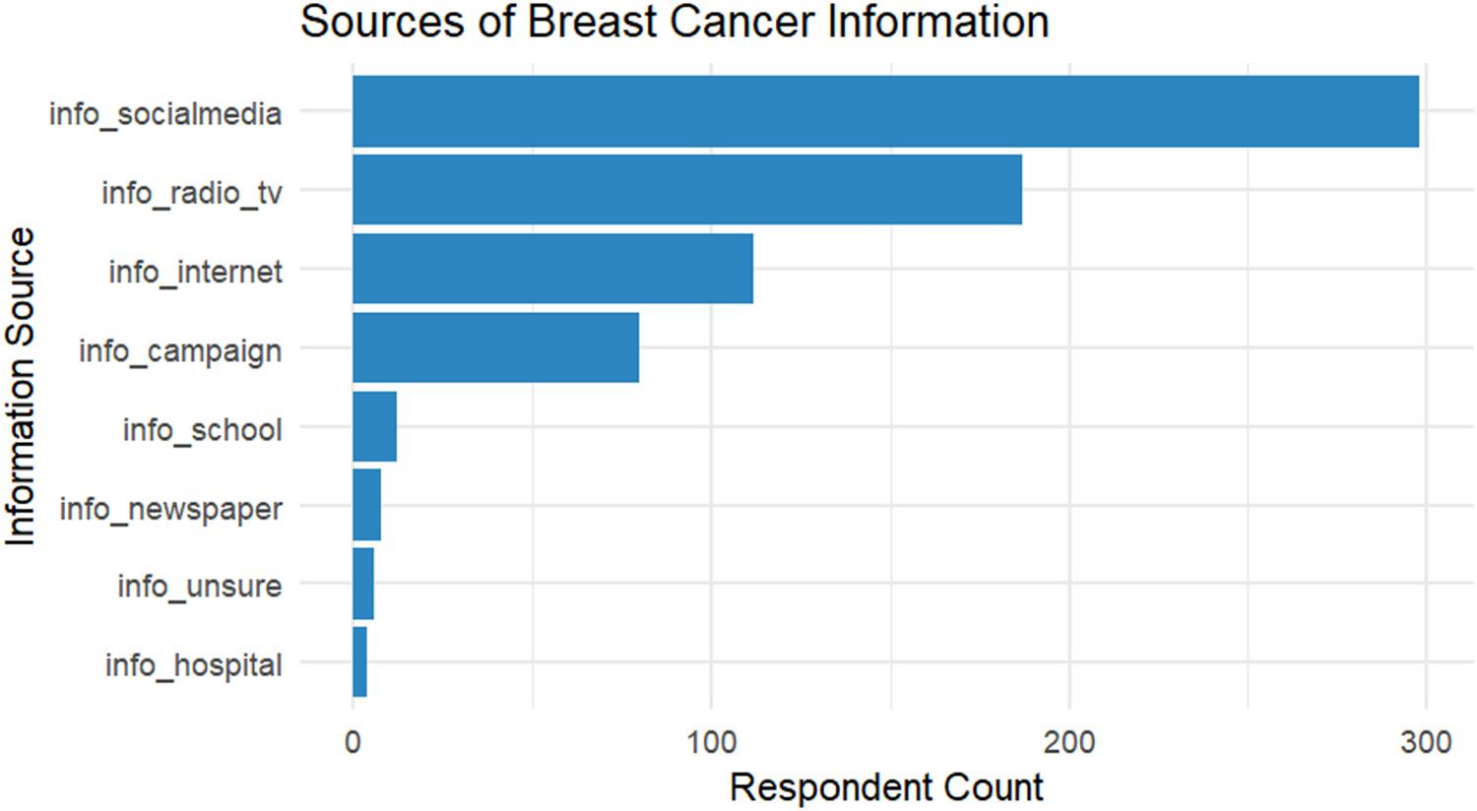
Distribution of primary information sources for BCa knowledge.

Information sources were reported by respondents through a multiple‐response item (Figure 3). Social media was the most frequently reported source (approximately 41.7%), followed by radio/television (26.2%), general internet sources (15.7%), and awareness campaigns (11.2%). Schools, hospitals, and newspapers were reported less frequently.

### 3.7 Impact of Information Source on BCa Knowledge

The multiple linear regression model examining information sources and knowledge scores was statistically significant (*p* < 0.001) (Table 13). In the fitted model, social media, radio/television, and awareness campaigns were associated with lower knowledge scores, with estimated differences of −1.56, −1.98, and −1.35 points, respectively. These are observational associations and cannot establish that the information source caused lower knowledge. Participants could also use multiple information sources, and people with lower knowledge may preferentially seek particular sources.

**Table 13:** Predictors of adequate BCa knowledge.

Predictor variable	Coefficient (β)	Standard error	*t*‐value	*p*‐value	Significance level
Intercept	4.32	0.49	8.87	< 0.001	***
Internet	0.39	0.51	0.76	0.446	ns
Social media	−1.56	0.49	−3.15	0.0017	**
Radio/Television	−1.98	0.5	−3.93	< 0.001	***
School	0.96	0.68	1.41	0.16	ns
Hospital	−0.31	0.95	−0.33	0.746	ns
Campaign	−1.35	0.51	−2.64	0.0084	**
Newspaper	−0.19	0.78	−0.25	0.804	ns
Not Sure	0.51	0.86	0.6	0.549	ns

*Note:* Binary logistic regression was used to estimate odds ratios (ORs) and 95% confidence intervals (CIs). The reference category for each predictor is indicated. Large ORs and wide confidence intervals reflect sparse data in some predictor categories and should be interpreted with caution.

#### 3.7.1 Information Sources and Willingness to Perform BSE

**Table 14:** Information sources and associations with knowledge and willingness to perform BSE.

Predictor variable	Coefficient (β)	Standard error	*z*‐value	*p*‐value	Significance level
Intercept	4.03	1.61	2.5	0.012	*
Internet	−0.72	1.64	−0.44	0.659	Ns
Social media	0.05	1.63	0.03	0.974	Ns
Radio/Television	−0.2	1.69	−0.12	0.903	Ns
School	14.42	1856.7	0.01	0.994	Ns
Hospital	14.16	3117.36	0.01	0.996	Ns
Campaign	−0.77	1.65	−4.18	0.64	Ns
Newspaper	14.54	2306.1	0.01	0.995	Ns
Not Sure	14.54	2662.86	0.01	0.996	Ns

*Note:* Some information source categories had small sample sizes, resulting in unstable estimates with large standard errors and wide confidence intervals. As shown in Table 14, none of the reported sources of breast cancer information was significantly associated with willingness to perform BSE.

Abbreviation: Ns, not statistically significant.

## 4 Discussion

This study examined BCa knowledge, attitudes, and willingness to perform BSE among adult females in Nigeria. The principal finding was a marked gap between broad awareness and actionable knowledge: although most respondents recognized BCa and expressed willingness to perform BSE, knowledge of specific risk factors, screening modalities, and the correct BSE procedure was substantially less complete. This finding is important because awareness of BCa does not necessarily translate into the knowledge, confidence, or skills required for effective preventive behavior. Previous studies in Nigeria and other sub‐Saharan African settings have similarly reported considerable variation in BCa knowledge and BSE practices, reflecting differences in study populations, education, geographical location, access to health information, and the methods used to define and measure “practice” [3, 5, 7, 13, 14, 16, 17].

The relatively high level of general awareness observed in the present study is consistent with previous Nigerian studies reporting substantial recognition of BCa among women [3, 5, 13]. However, the present findings also suggest that general awareness should not be equated with comprehensive understanding. Respondents were more likely to identify familiar risk factors, such as family history, obesity, and lifestyle‐related factors, than less commonly discussed factors related to reproductive history, breastfeeding, or other complex determinants. This pattern is consistent with previous Nigerian evidence showing that BCa awareness is often greater than detailed knowledge of its risk factors and early‐detection strategies [3, 13, 16]. One likely explanation is that public health messages tend to emphasize highly recognizable risk factors and common symptoms, whereas less obvious or non‐modifiable factors receive less attention in routine health education. In addition, the predominantly urban and highly educated nature of the present sample may have increased general exposure to BCa information without necessarily ensuring accurate or comprehensive knowledge.

The observed knowledge gap is particularly relevant because knowledge of BCa symptoms and risk factors may influence the interpretation of bodily changes and the decision to seek care. Previous studies among Nigerian women have identified inadequate knowledge, misconceptions, fear, and sociocultural factors as barriers to early presentation and diagnosis [4, 7, 8]. Similar barriers have been reported across sub‐Saharan Africa [14]. Thus, the present findings support the argument that BCa interventions should move beyond general awareness creation and provide accurate, practical information that enables women to recognize concerning symptoms and understand available screening and early‐detection options.

A major finding of this study was the discrepancy between a very high stated willingness to perform BSE and incomplete procedural knowledge. Nearly all respondents reported willingness to perform BSE after instruction, whereas less than half reported awareness of the BSE procedure. This finding should be interpreted carefully because the study measured willingness or intention to perform BSE rather than regular, technically correct, or directly observed BSE practice. Consequently, the high proportion reporting willingness should not be interpreted as evidence that BSE is widely or correctly practiced. Previous studies in Nigeria and other African settings have also reported substantial differences between awareness of BSE, knowledge of the correct procedure, and actual practice [5, 13, 17, 18, 19]. A review of African studies similarly found considerable variation in BSE awareness and practice across countries and populations [17].

The difference between the present findings and studies reporting lower BSE practice may partly reflect differences in the characteristics of the study populations. For example, studies conducted among rural women, market women, or women with lower educational attainment may identify lower levels of knowledge and practice because of reduced access to formal health information and screening services [7, 14, 20, 21]. In contrast, the present study recruited participants through an online, English‐language survey and included a predominantly urban and highly educated sample. Such participants may have greater exposure to BCa information and may therefore express greater willingness to perform BSE. However, willingness may not necessarily translate into sustained behavior. Previous research has demonstrated that the transition from health intention to actual preventive behavior can be influenced by self‐efficacy, practical skills, perceived barriers, social support, and reminders. Therefore, the gap observed in this study may represent an important distinction between being motivated to perform BSE and possessing the procedural competence and behavioral conditions required to perform it correctly.

The positive attitudes toward BCa screening observed in this study are broadly consistent with previous Nigerian research reporting favorable perceptions of early detection and BCa screening [5, 13, 16]. Most respondents recognized the importance of early detection and perceived screening as beneficial. However, the presence of misconceptions among a minority of respondents remains important. Even when the overall level of awareness is high, incorrect beliefs may discourage screening, delay help‐seeking, or reduce confidence in early detection. Previous studies have identified misconceptions and sociocultural beliefs as important influences on BCa‐related health behavior in Nigeria and other African settings [4, 8, 16]. The persistence of such beliefs may reflect inconsistent health messaging, exposure to inaccurate information, fear of cancer diagnosis, and culturally shaped interpretations of illness.

The association between education and willingness to perform BSE requires cautious interpretation. In the present study, higher educational attainment was associated with greater willingness to perform BSE, but education was not clearly associated with higher knowledge scores. This finding differs from the assumption that formal education necessarily translates into superior BCa knowledge. Previous studies have reported positive associations between education and BCa knowledge or preventive practices [13, 16, 18], although such associations have not been consistent across all populations [7, 14]. Several explanations are possible. First, formal education and health literacy are related but distinct constructs; an individual may have a high level of formal education but limited exposure to accurate BCa information. Second, the present sample was highly skewed towards participants with postgraduate education, while the basic‐education reference group was very small. This imbalance may have produced unstable estimates and distorted comparisons. Third, higher education may influence confidence or willingness to engage in preventive behavior without necessarily improving detailed knowledge of specific BSE procedures. The education‐related findings should therefore be regarded as exploratory rather than definitive.

Social media was the most commonly reported source of BCa information in this study. This finding reflects the increasing importance of digital platforms in health communication and is consistent with the growing use of online sources for health information among younger and urban populations. However, respondents who reported obtaining information from social media, radio/television, and campaigns had lower knowledge scores in the present analysis. This finding should not be interpreted as evidence that these sources cause poor knowledge. Participants could use multiple information sources, and people with different levels of knowledge may preferentially seek different types of information. Furthermore, the quality of information available through social media and mass media is highly variable. The finding may therefore reflect differences in the accuracy, completeness, or consistency of the information accessed rather than an inherent limitation of the communication channels themselves.

The present findings differ from studies that have reported beneficial associations between exposure to health information and BCa preventive behavior [10]. This difference may be explained by variation in how information exposure was measured. Some studies assess whether participants have ever received BCa information, whereas the present study examined specific reported information sources. Exposure to information does not necessarily indicate exposure to accurate, understandable, or actionable information. The findings therefore support a shift from simply increasing the volume of BCa information to improving its quality and ensuring that information is delivered through trusted and credible channels. Social media may be particularly valuable for reaching digitally connected women, but its use should be accompanied by evidence‐based content, professional oversight, and clear practical guidance.

The absence of a clear association between overall knowledge and willingness to perform BSE further suggests that knowledge alone may not be sufficient to produce preventive behavior. This finding is consistent with the broader KAP literature, which cautions against assuming that knowledge automatically leads to changes in attitudes or practices [22, 23]. The relationship between knowledge and behavior is often influenced by perceived susceptibility, perceived benefits, self‐efficacy, social norms, access to services, and practical barriers. In the present study, the outcome was willingness rather than verified BSE performance; therefore, the absence of an association should be interpreted narrowly. Nevertheless, the finding suggests that BCa interventions should combine information with practical demonstrations, opportunities for skill development, reinforcement, and behavioral support.

The study also has implications for the design of BCa awareness interventions in Nigeria. The findings suggest that broad awareness campaigns should be complemented by interventions that address the knowledge–practice gap. These may include demonstrations of BSE techniques, visual and digital educational materials, interactive community‐based education, and integration of breast health education into routine primary healthcare services. Previous research in Nigeria and other African settings suggests that interventions are more likely to be effective when they address the social and cultural context in which women make decisions about breast health [4, 8, 14, 20]. Digital platforms may provide an important opportunity to reach urban and educated women; however, complementary offline strategies remain essential for women who are rural, older, less educated, or digitally excluded.

The findings should be interpreted in light of several limitations. The cross‐sectional design prevents causal inference, and the online, English‐language, non‐probability recruitment strategy limits generalizability. The sample was predominantly urban and highly educated, and therefore may not represent Nigerian women with limited digital access or lower educational attainment. The study also relied on self‐reported responses, which may be affected by recall and social desirability bias. Most importantly, willingness to perform BSE should not be interpreted as evidence of regular or technically correct practice because actual performance was not observed or assessed using a validated behavioral measure. In addition, the sparse distribution of some predictor categories, particularly the small basic‐education reference group, resulted in imprecise and potentially unstable regression estimates. The regression analyses should therefore be interpreted as exploratory and hypothesis‐generating.

Despite these limitations, the study has important strengths. It included a relatively large sample and examined multiple dimensions of BCa awareness, including knowledge, attitudes, information sources, and willingness to engage in BSE. By examining the gap between broad awareness and actionable knowledge, the study provides a more nuanced understanding of BCa‐related health behavior than an assessment of awareness alone. The findings also contribute contemporary evidence on the role of digital and mass‐media information sources in BCa awareness among Nigerian women.

Overall, the present study demonstrates that high general awareness and positive attitudes do not necessarily translate into comprehensive knowledge or demonstrated preventive behavior. Among this digitally connected and relatively highly educated sample of adult Nigerian females, the most important gap was between willingness to perform BSE and knowledge of how BSE should be performed. Future interventions should therefore move beyond general awareness creation and prioritize accurate, practical, skill‐based education delivered through trusted and accessible channels. Further population‐based studies should examine whether similar knowledge–practice gaps exist among rural, older, less educated, and digitally excluded Nigerian women.

### 4.1 Strengths and Limitations

#### 4.1.1 Strength of Study

The strengths of this study include its relatively large sample size and its examination of multiple dimensions of BCa awareness, including knowledge, perceptions, information sources, and willingness to engage in BSE. By focusing on the gap between awareness and preventive intention, the study provides a more nuanced assessment of BCa health behavior than knowledge assessment alone. The findings also provide context‐specific evidence from Nigeria that may inform targeted BCa education and early‐detection interventions.

### 4.2 Study Limitations

This cross‐sectional study cannot establish causal relationships. The online, English‐language, non‐probability sampling approach may have introduced selection bias and limits generalizability, particularly to rural, less educated, older, and digitally excluded women. The findings are also based on self‐reported data, and willingness to perform BSE should not be interpreted as regular or technically correct BSE practice. Finally, imbalanced educational categories and sparse data may have contributed to unstable regression estimates, while associations between information sources and knowledge should be interpreted as correlational rather than causal.

## 5 Conclusion

This study found high general awareness of BCa among adult females in Nigeria, but important gaps in knowledge of its risk factors, symptoms, treatment, screening options, and the correct BSE procedure. Although willingness to perform BSE was high, this should not be interpreted as evidence of regular or technically correct BSE practice. The findings highlight the need for targeted, practical BCa education that goes beyond general awareness to improve procedural knowledge and confidence in early‐detection behaviors. Future interventions should use accurate, accessible, and culturally appropriate information delivered through trusted digital, community, and healthcare channels. Further population‐based research is needed to determine whether these knowledge–practice gaps persist among rural, older, less educated, and digitally underserved women in Nigeria.

## Author Contributions

**Madinat Hassan:** conceptualization, methodology, writing – review and editing, project administration, supervision, writing – original draft. **Taofeek Tope Adegboyega:** conceptualization; writing – original draft, writing – review and editing, methodology, formal analysis, supervision. **Bilkisu Usman Farouk:** writing – original draft, methodology, formal analysis, project administration. **Sunday Zeal Bala:** conceptualization, writing – original draft, writing – review and editing. **Rachael Olufunmilayo Oduyemi:** writing – review and editing, validation. **Kazeem Owolabi:** writing – review and editing, validation. **Munir Sani:** writing – review and editing, methodology, formal analysis, supervision. **Miracle Uwa Livinus:** formal analysis, data curation, visualization, validation, writing – original draft, software. **Hannatu Usman Ayuba:** methodology, supervision, resources. **Sadiq Hassan:** methodology, formal analysis, writing – review and editing. **Ifeyinwa Maureen Okeke:** writing – review and editing, software, data curation, resources. **Abednego Shekari:** methodology, investigation, software. **Murtala Jibril:** investigation, methodology, supervision, software.

## Artificial Intelligence Disclosure

Generative artificial intelligence tools were not used to conduct the study, collect or analyze data, or generate figures. Any language editing assistance used during manuscript preparation did not replace author responsibility for the content, interpretation, or final approval of the manuscript.

## Funding

The authors have nothing to report.

## Ethics Statement

This study was conducted in accordance with the ethical principles outlined in the Declaration of Helsinki (2013 revision), the Council for International Organizations of Medical Sciences (CIOMS) International Ethical Guidelines (2016), and the National Code of Health Research Ethics, Nigeria (2014). Ethical clearance was obtained from the Barau Dikko Teaching Hospital (BDTH) Human Research Ethical Committee (HREC) with registration number NHREC/30/11/21A. All participants were provided with information about the study objectives, and informed consent was obtained electronically before completing the questionnaire. Participation was entirely voluntary, and respondents were assured of the confidentiality and anonymity of their data.

## Consent

The authors have nothing to report.

## Conflicts of Interest

The authors declare no conflicts of interest.

## Transparency Statement

The lead/corresponding author, Madinat Hassan, affirms that this manuscript is an honest, accurate, and transparent account of the study being reported; that no important aspects of the study have been omitted; and that any discrepancies from the study as planned (and, if relevant, registered) have been explained.

## Data Availability

The data that support the findings of this study are available on request from the corresponding author. The data are not publicly available due to privacy or ethical restrictions. The datasets generated and/or analyzed during the current study are available from the corresponding author on reasonable request.
